# Detection of Congenital Mullerian Anomalies Using Real-Time 3D Sonography 

**Published:** 2011-09-23

**Authors:** Firoozeh Ahmadi, Hadieh Haghighi

**Affiliations:** Department of Reproductive Imaging, Reproductive Biomedicine Research Center, Royan Institute for Reproductive Biomedicine, ACECR, Tehran, Iran

**Figure:**
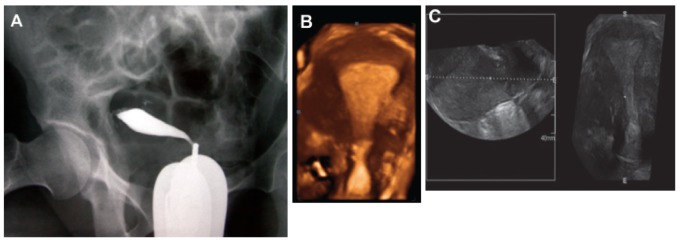


A 35 year-old woman referred to Royan Institute (Reproductive Biomedicine Research Center) for infertility treatment. She had an eleven-year history of primary infertility with a normal abdominal ultrasound. Hysterosalpingography (HSG) was obtained one month prior to referral in another center (Fig A).

The HSG finding of an apparent unicorn uterus followed by a normal vaginal ultrasound led us to perform a three-dimensional vaginal ultrasound before resorting to hysteroscopy. Results of the three-dimensional vaginal ultrasound revealed a normal uterus (Fig B, C).

Accurate characterization of congenital Mullerian anomalies (MDAs) such as an arcuate, unicornuate, didelphys, bicornuate or septate uterus is challenging. While HSG has been the standard test in the diagnosis of MDAs, some limitations may favor the use of three-dimensional ultrasound. The most difficult part of HSG is interpreting the two-dimensional radiographic image into a complex, three-dimensional living organ (1). A variety of technical problems may occur while performing HSG. In this case, only an oblique view could lead to a correct interpretation. It is advisable for the interpreter to perform the procedure rather than to inspect only the finished radiographic images (2).

One of the most useful scan planes obtained on three-dimensional ultrasound is the coronal view of the uterus. This view is known to be a valuable problem-solving tool that assists in differentiating between various types of MDAs due to the high level of agreement between three-dimensional ultrasound and HSG (3, 4).

Recently, three-dimensional ultrasound has become the sole mandatory step in the initial investigation of MDAs due to its superiority to other techniques that have been used for the same purpose (5).
